# Supplementary material for: Developing and Validating Multi-Modal Models for Mortality Prediction in COVID-19 Patients: a Multi-center Retrospective Study
Source: J Digit Imaging. 2022 Jul 5;35(6):1514–29. doi: 10.1007/s10278-022-00674-z (PMC9255527; doi:10.1007/s10278-022-00674-z)
Supplement: Supplementary file 1 — Supplementary file1 (DOCX 58 KB) [file 10278_2022_674_MOESM1_ESM.docx]

# **Supplementary Material**

**Part 1 - CLAIM: Checklist for Artificial Intelligence in Medical Imaging**[1]

| **Section / Topic** | **No.** | **Item** | **Done** |
| --- | --- | --- | --- |
| TITLE / ABSTRACT |  |  |  |
|  | 1 | Identification as a study of AI methodology, specifying the category of technology used (e.g., deep learning) | yes |
|  | 2 | Structured summary of study design, methods, results, and conclusions | n/a |
| INTRODUCTION |  |  |  |
|  | 3 | Scientific and clinical background, including the intended use and clinical role of the AI approach | yes |
|  | 4 | Study objectives and hypotheses | yes |
| METHODS |  |  |  |
| *Study Design* | 5 | Prospective or retrospective study | yes |
|  | 6 | Study goal, such as model creation, exploratory study, feasibility study, non-inferiority trial | yes |
| *Data* | 7 | Data sources | yes |
|  | 8 | Eligibility criteria: how, where, and when potentially eligible participants or studies were identified (e.g., symptoms, results from previous tests, inclusion in registry, patient-care setting, location, dates) | yes |
|  | 9 | Data pre-processing steps | yes |
|  | 10 | Selection of data subsets, if applicable | yes |
|  | 11 | Definitions of data elements, with references to Common Data Elements | yes |
|  | 12 | De-identification methods | yes |
|  | 13 | How missing data were handled | yes |
| *Ground Truth* | 14 | Definition of ground truth reference standard, in sufficient detail to allow replication | yes |
|  | 15 | Rationale for choosing the reference standard (if alternatives exist) | yes |
|  | 16 | Source of ground-truth annotations; qualifications and preparation of annotators | n/a |
|  | 17 | Annotation tools | yes |
|  | 18 | Measurement of inter- and intra-rater variability; methods to mitigate variability and/or resolve discrepancies | n/a |
| *Data Partitions* | 19 | Intended sample size and how it was determined | n/a |
|  | 20 | How data were assigned to partitions; specify proportions | yes |
|  | 21 | Level at which partitions are disjoint (e.g., image, study, patient, institution) | yes |
| *Model* | 22 | Detailed description of model, including inputs, outputs, all intermediate layers and connections | yes |
|  | 23 | Software libraries, frameworks, and packages | yes |
|  | 24 | Initialization of model parameters (e.g., randomization, transfer learning) | yes |
| *Training* | 25 | Details of training approach, including data augmentation, hyperparameters, number of models trained | yes |
|  | 26 | Method of selecting the final model | yes |
|  | 27 | Ensembling techniques, if applicable | yes |
| *Evaluation* | 28 | Metrics of model performance | yes |
|  | 29 | Statistical measures of significance and uncertainty (e.g., confidence intervals) | yes |
|  | 30 | Robustness or sensitivity analysis | yes |
|  | 31 | Methods for explainability or interpretability (e.g., saliency maps), and how they were validated | yes |
|  | 32 | Validation or testing on external data | yes |
| RESULTS |  |  |  |
| *Data* | 33 | Flow of participants or cases, using a diagram to indicate inclusion and exclusion | yes |
|  | 34 | Demographic and clinical characteristics of cases in each partition | yes |
| *Model performance* | 35 | Performance metrics for optimal model(s) on all data partitions | yes |
|  | 36 | Estimates of diagnostic accuracy and their precision (such as 95% confidence intervals) | yes |
|  | 37 | Failure analysis of incorrectly classified cases | no |
| DISCUSSION |  |  |  |
|  | 38 | Study limitations, including potential bias, statistical uncertainty, and generalizability | yes |
|  | 39 | Implications for practice, including the intended use and/or clinical role | yes |
| OTHER INFORMATION |  |  |  |
|  | 40 | Registration number and name of registry | n/a |
|  | 41 | Where the full study protocol can be accessed | n/a |
|  | 42 | Sources of funding and other support; role of funders | yes |

**Part 2 - Detailed patient characteristics tables**

For statistical analysis, hypothesis test functions used by default are chi-square test for categorical variables (with continuity correction) and one-way test for continuous variables (with equal variance assumption, i.e., regular ANOVA). Two-group ANOVA is equivalent to the t-test. Statistical analyses were performed in Python version 3.7.10. All tests were two-sided, with a 0.05 level of significance.

**Supplementary Table 1. Patient Characteristics for the Madrid dataset**

|  |  | **Missing** | **Overall** | **Alive** | **Expired** | **P-Value** |
| --- | --- | --- | --- | --- | --- | --- |
| n |  |  | 1628 | 1439 | 189 |  |
| Age, mean (SD) |  | 0 | 67.3 (15.8) | 65.7 (15.7) | 79.6 (10.0) | <0.001 |
| Sex (%) | FEMALE | 0 | 648 (39.8) | 594 (41.3) | 54 (28.6) | <0.001 |
| Temperature (SD) |  | 388 | 36.8 (0.8) | 36.8 (0.8) | 36.8 (0.9) | 0.698 |
| Systolic BP (SD) |  | 604 | 131.0 (21.4) | 131.1 (20.8) | 130.8 (25.1) | 0.916 |
| Diastolic BP (SD) |  | 600 | 75.9 (34.2) | 76.3 (36.2) | 73.2 (14.1) | 0.072 |
| Heart Rate (SD) |  | 368 | 89.7 (16.4) | 89.8 (16.4) | 89.0 (16.4) | 0.558 |
| O2 Saturation (SD) |  | 354 | 92.7 (6.6) | 93.3 (5.8) | 88.3 (9.8) | <0.001 |
| Mortality Outcome, n (%) | FALSE | 0 | 1439 (88.4) | 1439 (100.0) |  | <0.001 |
|  | TRUE |  | 189 (11.6) |  | 189 (100.0) |  |
| Diabetes, n (%) | 1.0 | 9 | 1344 (83.0) | 1202 (83.9) | 142 (75.9) | 0.008 |
| Hyperlipidemia, n (%) | 1.0 | 9 | 439 (27.1) | 373 (26.0) | 66 (35.3) | 0.010 |
| Hypertension, n (%) | 1.0 | 9 | 107 (6.6) | 88 (6.1) | 19 (10.2) | 0.055 |
| Ischemic Heart Disease, n (%) | 1.0 | 9 | 111 (6.9) | 86 (6.0) | 25 (13.4) | <0.001 |
| Chronic Kidney Disease, n (%) | 1.0 | 9 | 95 (5.9) | 73 (5.1) | 22 (11.8) | <0.001 |
| Chronic Obstructive Pulmonary Disease, n (%) | 1.0 | 9 | 83 (5.1) | 68 (4.7) | 15 (8.0) | 0.083 |
| Bronchial Asthma, n (%) | 1.0 | 9 | 83 (5.1) | 75 (5.2) | 8 (4.3) | 0.702 |
| Active Cancer, n (%) | 1.0 | 9 | 80 (4.9) | 57 (4.0) | 23 (12.3) | <0.001 |
| Chronic Liver Disease, n (%) | 1.0 | 9 | 18 (1.1) | 9 (0.6) | 9 (4.8) | <0.001 |
| Previous Stroke, n (%) | 1.0 | 9 | 51 (3.2) | 37 (2.6) | 14 (7.5) | <0.001 |
| Congestive Heart Failure, n (%) | 1.0 | 9 | 75 (4.6) | 62 (4.3) | 13 (7.0) | 0.156 |
| Dementia, n (%) | 1.0 | 9 | 52 (3.2) | 40 (2.8) | 12 (6.4) | 0.015 |
| LDH, mean (SD) |  | 388 | 594.5 (330.8) | 568.1 (257.2) | 805.9 (638.2) | <0.001 |
| MCV, mean (SD) |  | 318 | 88.0 (5.5) | 87.8 (5.3) | 89.4 (6.5) | 0.003 |
| Neutrophil %, mean (SD) |  | 319 | 72.8 (12.1) | 72.1 (11.9) | 78.2 (12.3) | <0.001 |
| aPTT, mean (SD) |  | 769 | 33.0 (6.8) | 32.9 (6.6) | 33.7 (8.1) | 0.359 |
| D-dimer, mean (SD) |  | 600 | 1789.2 (4490.1) | 1694.7 (4448.3) | 2620.6 (4784.2) | 0.061 |
| INR, mean (SD) |  | 641 | 1.4 (1.2) | 1.4 (1.1) | 1.5 (1.3) | 0.392 |
| Glucose, mean (SD) |  | 389 | 125.7 (43.3) | 123.0 (38.5) | 146.1 (66.1) | <0.001 |
| Blood Urea Nitrogen, mean (SD) |  | 367 | 42.4 (32.8) | 39.0 (25.8) | 67.7 (58.7) | <0.001 |
| Lymphocyte %, mean (SD) |  | 319 | 18.4 (9.7) | 18.9 (9.6) | 14.1 (9.4) | <0.001 |
| MCH, mean (SD) |  | 318 | 29.6 (2.0) | 29.6 (2.0) | 29.9 (2.3) | 0.124 |
| AST, mean (SD) |  | 461 | 43.7 (33.2) | 42.7 (33.2) | 51.0 (32.1) | 0.005 |
| RDW, mean (SD) |  | 327 | 12.8 (2.1) | 12.7 (2.0) | 13.5 (2.3) | <0.001 |
| RBC count, mean (SD) |  | 318 | 4.7 (0.6) | 4.7 (0.6) | 4.6 (0.7) | 0.073 |
| Neutrophil, mean (SD) |  | 318 | 5.4 (3.3) | 5.2 (2.9) | 7.0 (5.0) | <0.001 |
| Hemoglobin, mean (SD) |  | 318 | 13.8 (1.8) | 13.8 (1.8) | 13.6 (2.2) | 0.352 |
| CRP, mean (SD) |  | 329 | 92.8 (89.5) | 87.3 (85.9) | 136.2 (104.5) | <0.001 |
| ALT, mean (SD) |  | 480 | 37.7 (35.2) | 38.1 (35.8) | 34.6 (30.8) | 0.220 |
| Creatinine, mean (SD) |  | 337 | 1.0 (0.6) | 0.9 (0.6) | 1.3 (0.8) | <0.001 |
| Mean Platelet Volume, mean (SD) |  | 332 | 10.3 (1.0) | 10.3 (1.0) | 10.6 (1.0) | <0.001 |
| Platelet, mean (SD) |  | 318 | 217.1 (94.7) | 219.8 (92.2) | 197.0 (110.0) | 0.015 |
| Prothrombin Activity, mean (SD) |  | 641 | 74.5 (16.8) | 74.7 (16.5) | 72.8 (18.9) | 0.296 |
| Leukocyte, mean (SD) |  | 318 | 7.1 (3.6) | 6.9 (3.4) | 8.4 (4.7) | <0.001 |
| Serum Sodium, mean (SD) |  | 346 | 136.9 (4.5) | 136.8 (4.0) | 137.5 (7.3) | 0.292 |
| Lymphocyte, mean (SD) |  | 319 | 1.2 (1.3) | 1.2 (1.3) | 0.9 (0.5) | <0.001 |
| Hematocrit, mean (SD) |  | 318 | 40.9 (5.0) | 40.9 (4.8) | 40.8 (6.0) | 0.831 |
| Serum Potassium, mean (SD) |  | 360 | 4.2 (0.5) | 4.2 (0.5) | 4.3 (0.7) | 0.323 |

LDH: lactate dehydrogenase, MCV: mean corpuscular volume, aPTT: partial thromboplastin time, INR: international normalized ratio, MCH: mean corpuscular hemoglobin , AST: aspartate transaminase, RDW: red cell distribution width, RBC: red blood cell, CRP: C-reactive protein, ALT: alanine transaminase

**Supplementary Table 2. Patient characteristics in Hoboken and Seoul Cohorts**

|  |  | **Hoboken University Medical Center** | | | | **Seoul National University Hospital** | | | |
| --- | --- | --- | --- | --- | --- | --- | --- | --- | --- |
|  |  | Overall | Alive | Expired | p-value | Overall | Alive | Expired | p-value |
| n |  | 201 | 114 | 87 |  | 315 | 310 | 5 |  |
| Age, mean (SD) |  | 65.0 (16.9) | 61.9 (16.5) | 69.1 (16.5) | 0.003 | 46.0 (19.6) | 45.7 (19.6) | 64.0 (15.2) | 0.053 |
| Sex (%) | F | 83 (41.3) | 55 (48.2) | 28 (32.2) | 0.032 | 150 (47.6) | 150 (48.4) |  | 0.062 |
| Temperature (SD) |  | 38.0 (0.9) | 38.0 (0.9) | 38.0 (0.9) | 0.853 | 36.7 (0.7) | 36.7 (0.7) | 37.0 (0.6) | 0.320 |
| Systolic BP (SD) |  | 129.6 (24.0) | 131.9 (23.1) | 126.5 (25.0) | 0.120 | 122.6 (18.5) | 122.1 (18.1) | 151.6 (22.0) | 0.039 |
| Diastolic BP (SD) |  | 73.9 (14.1) | 75.7 (13.9) | 71.5 (13.9) | 0.033 | 86.3 (26.0) | 85.9 (26.9) | 91.4 (9.0) | 0.323 |
| Heart Rate (SD) |  | 105.0 (19.7) | 103.8 (16.9) | 106.6 (22.9) | 0.345 | 96.7 (2.5) | 96.8 (2.4) | 92.8 (5.4) | 0.176 |
| O2 Saturation (SD) |  | 86.7 (12.5) | 91.1 (8.2) | 81.0 (14.7) | <0.001 | 96.7 (2.5) | 96.8 (2.4) | 92.8 (5.4) | 0.176 |
| Mortality Outcome, n (%) | 0 | 114 (56.7) | 114 (100.0) |  | <0.001 | 310 (98.4) | 310 (100.0) |  | <0.001 |
|  | 1 | 87 (43.3) |  | 87 (100.0) |  | 5 (1.6) |  | 5 (100.0) |  |
| Diabetes, n (%) | 1 | 76 (37.8) | 45 (39.5) | 31 (35.6) | 0.682 | 36 (11.5) | 34 (11.0) | 2 (40.0) | 0.103 |
| Hyperlipidemia, n (%) | 1 | 67 (33.3) | 37 (32.5) | 30 (34.5) | 0.880 | 20 (6.4) | 19 (6.2) | 1 (20.0) | 0.283 |
| Hypertension, n (%) | 1 | 110 (54.7) | 62 (54.4) | 48 (55.2) | 0.974 | 47 (15.0) | 43 (14.0) | 4 (80.0) | 0.002 |
| Ischemic Heart Disease, n (%) | 1 | 32 (15.9) | 19 (16.7) | 13 (14.9) | 0.891 | 8 (2.6) | 7 (2.3) | 1 (20.0) | 0.122 |
| Chronic Kidney Disease, n (%) | 1 | 26 (12.9) | 8 (7.0) | 18 (20.7) | 0.008 | 5 (1.6) | 4 (1.3) | 1 (20.0) | 0.078 |
| COPD, n (%) | 1 | 20 (10.0) | 11 (9.6) | 9 (10.3) | 0.941 | 3 (1.0) | 2 (0.6) | 1 (20.0) | 0.047 |
| Bronchial Asthma, n (%) | 1 | 25 (12.4) | 19 (16.7) | 6 (6.9) | 0.062 | 2 (0.6) | 2 (0.6) |  | 1.000 |
| Active Cancer, n (%) | 1 | 10 (5.0) | 7 (6.1) | 3 (3.4) | 0.519 |  |  |  |  |
| CLD, n (%) | 1 | 1 (0.5) | 1 (0.9) |  | 1.000 | 3 (1.0) | 3 (1.0) |  | 1.000 |
| Previous Stroke, n (%) | 1 | 7 (3.5) | 3 (2.6) | 4 (4.6) | 0.469 | 8 (2.6) | 8 (2.6) |  | 1.000 |
| Congestive Heart Failure, n (%) | 1 | 32 (15.9) | 19 (16.7) | 13 (14.9) | 0.891 | 6 (1.9) | 5 (1.6) | 1 (20.0) | 0.093 |
| Dementia, n (%) | 1 | 28 (13.9) | 9 (7.9) | 19 (21.8) | 0.009 | 3 (1.0) | 3 (1.0) |  | 1.000 |
| LDH, mean (SD) |  | 1324.4 (1756.1) | 957.1 (372.6) | 1768.9 (2515.5) | 0.005 | 0.0 (0.0) | 0.0 (0.0) | 0.0 (0.0) | nan |
| MCV, mean (SD) |  | 87.5 (5.6) | 87.3 (5.5) | 87.9 (5.7) | 0.417 | 0.0 (0.0) | 0.0 (0.0) | 0.0 (0.0) | nan |
| Neutrophil %, mean (SD) |  | 82.1 (30.9) | 75.6 (15.3) | 89.9 (41.6) | 0.003 | 0.0 (0.0) | 0.0 (0.0) | 0.0 (0.0) | nan |
| aPTT, mean (SD) |  | 33.3 (5.7) | 32.6 (4.4) | 34.0 (6.8) | 0.110 | 0.0 (0.0) | 0.0 (0.0) | 0.0 (0.0) | nan |
| D-dimer, mean (SD) |  | 0.0 (0.0) | 0.0 (0.0) | 0.0 (0.0) |  | 0.0 (0.0) | 0.0 (0.0) | 0.0 (0.0) | nan |
| INR, mean (SD) |  | 1.2 (0.2) | 1.2 (0.2) | 1.2 (0.2) | 0.035 | 0.0 (0.0) | 0.0 (0.0) | 0.0 (0.0) | nan |
| Glucose, mean (SD) |  | 165.9 (90.1) | 153.6 (86.5) | 180.7 (92.5) | 0.039 | 0.0 (0.0) | 0.0 (0.0) | 0.0 (0.0) | nan |
| Blood Urea Nitrogen, mean (SD) |  | 31.8 (29.7) | 23.3 (21.9) | 42.2 (34.4) | <0.001 | 0.0 (0.0) | 0.0 (0.0) | 0.0 (0.0) | nan |
| Lymphocyte %, mean (SD) |  | 12.5 (10.8) | 14.6 (10.7) | 10.0 (10.3) | 0.003 | 0.0 (0.0) | 0.0 (0.0) | 0.0 (0.0) | nan |
| MCH, mean (SD) |  | 28.8 (2.2) | 28.8 (2.2) | 28.9 (2.3) | 0.826 | 0.0 (0.0) | 0.0 (0.0) | 0.0 (0.0) | nan |
| AST, mean (SD) |  | 102.1 (252.0) | 74.3 (70.6) | 135.7 (364.6) | 0.125 | 0.0 (0.0) | 0.0 (0.0) | 0.0 (0.0) | nan |
| RDW, mean (SD) |  | 14.8 (2.0) | 14.5 (1.9) | 15.1 (2.0) | 0.045 | 0.0 (0.0) | 0.0 (0.0) | 0.0 (0.0) | nan |
| RBC count, mean (SD) |  | 4.6 (0.7) | 4.6 (0.6) | 4.6 (0.7) | 0.674 | 0.0 (0.0) | 0.0 (0.0) | 0.0 (0.0) | nan |
| Neutrophil, mean (SD) |  | 8.4 (8.7) | 6.7 (4.2) | 10.5 (11.8) | 0.005 | 0.0 (0.0) | 0.0 (0.0) | 0.0 (0.0) | nan |
| Hemoglobin, mean (SD) |  | 14.0 (10.4) | 13.4 (1.9) | 14.8 (15.3) | 0.378 | 0.0 (0.0) | 0.0 (0.0) | 0.0 (0.0) | nan |
| CRP, mean (SD) |  | 166.4 (120.3) | 114.2 (90.9) | 231.5 (121.1) | <0.001 | 0.0 (0.0) | 0.0 (0.0) | 0.0 (0.0) | nan |
| ALT, mean (SD) |  | 66.1 (149.1) | 52.2 (62.9) | 82.6 (208.9) | 0.193 | 0.0 (0.0) | 0.0 (0.0) | 0.0 (0.0) | nan |
| Creatinine, mean (SD) |  | 2.0 (3.7) | 1.3 (2.2) | 2.7 (4.9) | 0.019 | 0.0 (0.0) | 0.0 (0.0) | 0.0 (0.0) | nan |
| Mean Platelet Volume, mean (SD) |  | 8.5 (1.0) | 8.4 (0.9) | 8.5 (1.0) | 0.499 | 0.0 (0.0) | 0.0 (0.0) | 0.0 (0.0) | nan |
| Platelet, mean (SD) |  | 242.3 (96.6) | 240.6 (89.4) | 244.4 (105.1) | 0.793 | 0.0 (0.0) | 0.0 (0.0) | 0.0 (0.0) | nan |
| Prothrombin Activity, mean (SD) |  | 13.8 (2.2) | 13.5 (2.1) | 14.2 (2.4) | 0.093 | 0.0 (0.0) | 0.0 (0.0) | 0.0 (0.0) | nan |
| Leukocyte, mean (SD) |  | 10.0 (8.6) | 9.0 (8.5) | 11.1 (8.8) | 0.095 | 0.0 (0.0) | 0.0 (0.0) | 0.0 (0.0) | nan |
| Serum Sodium, mean (SD) |  | 136.7 (7.0) | 135.3 (4.6) | 138.5 (8.8) | 0.003 | 0.0 (0.0) | 0.0 (0.0) | 0.0 (0.0) | nan |
| Lymphocyte, mean (SD) |  | 1.3 (5.0) | 1.6 (6.7) | 1.0 (1.3) | 0.338 | 0.0 (0.0) | 0.0 (0.0) | 0.0 (0.0) | nan |
| Hematocrit, mean (SD) |  | 40.4 (5.7) | 40.5 (5.5) | 40.3 (6.0) | 0.870 | 0.0 (0.0) | 0.0 (0.0) | 0.0 (0.0) | nan |
| Serum Potassium, mean (SD) |  | 4.7 (6.6) | 5.0 (8.9) | 4.3 (0.6) | 0.364 | 0.0 (0.0) | 0.0 (0.0) | 0.0 (0.0) | nan |

COPD: Chronic Obstructive Pulmonary Disease, CLD: Chronic Liver Disease, LDH: lactate dehydrogenase, MCV: mean corpuscular volume, aPTT: partial thromboplastin time, INR: international normalized ratio, MCH: mean corpuscular hemoglobin , AST: aspartate transaminase, RDW: red cell distribution width, RBC: red blood cell, CRP: C-reactive protein, ALT: alanine transaminase

**Part 3 – Training and model picking**

**Supplementary Table 3. Structured EHR-based model’s hyperparameter search space**

|  | **Hyperparameter** | **Choices** | **No. of choices** |
| --- | --- | --- | --- |
| Logistic regression* | Alpha | uniform(0.0001,0.001) | range |
|  | Penalty | [l1, l2, 'elasticnet’] | 3 |
|  | l1_ratio | uniform(0.01,0.30) | range |
| Random forest | bootstrap | [True, False] | 2 |
|  | max_depth | randint(3,12) | 10 |
|  | max_features | ['auto', 'sqrt'] | 2 |
|  | min_samples_split | randint(2,12) | 11 |
|  | min_samples_leaf | randint(2,12) | 11 |
|  | n_estimators | randint(200, 1000) | 801 |
| Gradient boosting | Loss | ['deviance','exponential'] | 2 |
|  | learning_rate | uniform(0.003, 0.3) | range |
|  | n_estimators | randint(200, 1000) | 801 |
|  | subsample | uniform(0.1, 1) | range |
|  | criterion | ['friedman_mse','mse','mae'] | 3 |
|  | min_samples_split | randint(2,12) | 11 |
|  | min_samples_leaf' | randint(2,12) | 11 |
|  | max_depth | randint(3,12) | 10 |
|  | max_features | ['sqrt', 'log2'] | 2 |
| XGBoosting | colsample_bytree | uniform(0.1,1) | range |
|  | eta | (0.0001,0.1) | 2 |
|  | max_depth | randint(3,12) | 10 |
|  | min_child_weight | randint(3,12) | 10 |
|  | subsample | uniform(0.1,1) | range |

Note: *: The best model were selected with hyperparameters Alpha = 0.0007, Penalty = l1, and l1 ratio = 0.02 for EHR-based model; Alpha = 0.0005, Penalty = l1, and l1 ratio = 0.255 for fusion model.

**Supplementary Table 4. CXR-based model’s hyperparameter search space**

| **Hyperparameter** | **Choices** | **No. of choices** | **Best param** |
| --- | --- | --- | --- |
| Bbox augmentation | [True, False] | 2 | True |
| Model teacher | [224x224 CheXNet model, 320x320 MIMIC model][2] | 2 | 320x320 MIMIC model |
| Frozen layers | [0-355, 0-400, 0-420] | 3 | 0-400 |
| Feature layer | [-2, -4] | 2 | -2 |
| Hidden layers for classifier | [None, [512], [128, 128], [128, 64], [128]] | 5 | [128, 64] |
| Activation function for classifier | [ReLU, LeakyReLU] | 2 | LeakyReLU |
| Output initial bias | [None, calculated initial bias (np.log([expired/alive])] | 2 | Calculated initial bias |
| Batch size | [16, 32] | 2 | 16 |
| Class weight | [1:10, 3:10, 5:10, 8:10, 1:1, calculated weights of 0.56:4.47] | 6 | 1:1 (i.e., no re-weighting) |
| Drop out | [0.1, 0.25, 0.3, 0.4, 0.5] | 5 | 0.5 |
| Initial learning rate (with a fixed exponential decay rate of 0.96) | [0.001, 0.002, 0.003, 0.005, 0.01, 0.015] | 6 | 0.002 |
| Number of epochs | Chosen by early stopping with patience of 2 or 5 epochs up to maximum of 30 epochs (fixed) | 2 | 19 epochs with patience of 2 |

**Part 4 - Fairness analysis details**

**Supplementary Table 5. Evaluation metrics of fairness analysis between male and female patients.**

|  |  |  | **Male** | **Female** |
| --- | --- | --- | --- | --- |
| **Madrid** | EHR-based | AUROC | 0.83 [0.80-0.86] | 0.77 [0.72-0.82] |
|  |  | Sensitivity | 0.81 [0.73-0.86] | 0.72 [0.61-0.82] |
|  |  | Specificity | 0.71 [0.66-0.78] | 0.66 [0.57-0.76] |
|  |  | PPV | 0.29 [0.25-0.34] | 0.15 [0.11-0.2] |
|  |  | NPV | 0.96 [0.95-0.97] | 0.97 [0.95-0.98] |
|  |  | F1-score | 0.43 [0.38-0.47] | 0.24 [0.19-0.3] |
|  |  | Accuracy | 0.72 [0.68-0.77] | 0.66 [0.58-0.75] |
|  | CXR-based | AUROC | 0.79 [0.76-0.82] | 0.81 [0.78-0.83] |
|  |  | Sensitivity | 0.75 [0.68-0.83] | 0.76 [0.71-0.82] |
|  |  | Specificity | 0.71 [0.64-0.76] | 0.72 [0.67-0.75] |
|  |  | PPV | 0.28 [0.23-0.33] | 0.25 [0.21-0.28] |
|  |  | NPV | 0.95 [0.94-0.96] | 0.96 [0.95-0.97] |
|  |  | F1-score | 0.40 [0.35-0.45] | 0.37 [0.33-0.41] |
|  |  | Accuracy | 0.72 [0.66-0.76] | 0.73 [0.68-0.76] |
|  | Fusion | AUROC | 0.84 [0.81-0.86] | 0.85 [0.81-0.88] |
|  |  | Sensitivity | 0.78 [0.72-0.84] | 0.79 [0.71-0.87] |
|  |  | Specificity | 0.74 [0.68-0.79] | 0.76 [0.69-0.82] |
|  |  | PPV | 0.31 [0.26-0.36] | 0.21 [0.16-0.27] |
|  |  | NPV | 0.96 [0.95-0.97] | 0.98 [0.97-0.99] |
|  |  | F1-score | 0.44 [0.39-0.49] | 0.33 [0.26-0.41] |
|  |  | Accuracy | 0.75 [0.70-0.78] | 0.76 [0.70-0.82] |
| **Hoboken** | EHR-based | AUROC | 0.74 [0.67-0.82] | 0.73 [0.62-0.83] |
|  |  | Sensitivity | 0.68 [0.57-0.79] | 0.67 [0.52-0.81] |
|  |  | Specificity | 0.73 [0.62-0.84] | 0.71 [0.58-0.84] |
|  |  | PPV | 0.72 [0.62-0.83] | 0.55 [0.40-0.71] |
|  |  | NPV | 0.70 [0.60-0.79] | 0.81 [0.71-0.9] |
|  |  | F1-score | 0.70 [0.61-0.78] | 0.60 [0.48-0.71] |
|  |  | Accuracy | 0.71 [0.64-0.77] | 0.70 [0.60-0.78] |
|  | CXR-based | AUROC | 0.77 [0.69-0.84] | 0.66 [0.55-0.76] |
|  |  | Sensitivity | 0.76 [0.62-0.9] | 0.65 [0.48-0.85] |
|  |  | Specificity | 0.70 [0.58-0.82] | 0.64 [0.44-0.82] |
|  |  | PPV | 0.72 [0.62-0.81] | 0.49 [0.35-0.65] |
|  |  | NPV | 0.74 [0.63-0.88] | 0.78 [0.67-0.88] |
|  |  | F1-score | 0.74 [0.65-0.81] | 0.55 [0.44-0.66] |
|  |  | Accuracy | 0.73 [0.66-0.8] | 0.64 [0.54-0.75] |
|  | Fusion | AUROC | 0.75 [0.67-0.82] | 0.69 [0.57-0.79] |
|  |  | Sensitivity | 0.66 [0.53-0.8] | 0.65 [0.50-0.8] |
|  |  | Specificity | 0.75 [0.58-0.9] | 0.70 [0.56-0.84] |
|  |  | PPV | 0.73 [0.61-0.87] | 0.53 [0.38-0.69] |
|  |  | NPV | 0.68 [0.59-0.78] | 0.80 [0.70-0.89] |
|  |  | F1-score | 0.69 [0.60-0.77] | 0.58 [0.46-0.69] |
|  |  | Accuracy | 0.70 [0.64-0.77] | 0.68 [0.59-0.77] |

Note: No cases of death amongst females in the Seoul dataset.

**Part 5 - Project code and resources**

The authors provided open access to all their data extraction, filtering, data wrangling, modeling, figures and tables, code, and queries on <https://github.com/theonesp/multimodal_mortality_covid> . The de-identified version of Madrid COVID Data Saves Lives repository can be requested at <https://www.hmhospitales.com/coronavirus/covid-data-save-lives/english-version>

**Supplementary Table 6. Code and other resources made available to the community from work presented in this paper.**

| **Folder** | **Notebook** | **Content** |
| --- | --- | --- |
| 1.cxr_wrangling | hoboken_cxr_features_model.ipynb | In this notebook 320x320 CXR jpg and labels are read from Hoboken CXRs. The model previously trained to classify mortality on the Madrid CXRs is used to extract 64 features and output the prediction from the Hoboken CXR.  This code is written following a federated approach so only users with Hoboken credentials interact with the CXrs and only needs the *.jpg(s) to be located in their Google Drive. |
|  | seoul_cxr_features_model.ipynb | In this notebook 320x320 CXR jpg and labels are read from Seoul CXRs. The model previously trained to classify mortality on the Madrid CXR is used to extract 64 features and output the prediction from the Seoul CXR. |
|  | madrid_cxr_augmentation_w_bboxes.ipynb | In this notebook CXR augmentation with bboxes scratch is created, both image and tabular data are loaded in synch with each other, finally iterable loaders that can have the output X1, X2, y for enumerate in order to locate the bbox trachea and lung/heart coordinates. |
|  | useful_dicom_metadata.ipynb | In this notebook useful function examples from the pydicom library for exporting selected DICOM metadata into a txt, counting by group and basic QC tests after data conversion from DICOM to jpg. |
| 2.ehr_data_wrangling | madrid_ehr.ipynb | In this notebook Madrid tables are ingested; data is explored; variables are renamed or created; exclusion criteria is applied; vitals, comorbidities, drugs and labs are appropriately transformed and cleaned; tables are joined; table 1 is produced; feature distribution is evaluated; dataset is split into training and internal validation; model is trained with ehr data, evaluated using 4 folds cross-validation and calibrated; model is externally evaluated with other datasets and feature importance is addressed. |
|  | hoboken_ehr.ipynb | In this notebook we are preparing the Hoboken test dataset: Hoboken tables are ingested; exclusion criteria is applied; data is explored; vitals, comorbidities, drugs and labs are appropriately transformed and cleaned; variables are mean centered and standardized; missing values are imputed; table 1 is produced; image features are appended and renamed and tables are joined. |
|  | seoul_ehr.ipynb | In this notebook we are preparing the Seoul test dataset:  Seoul tables are ingested; exclusion criteria are applied; data is explored; vitals, comorbidities, drugs and labs are appropriately transformed and cleaned; variables are mean centered and standardized; missing values are imputed; table 1 is produced; image features are appended and renamed and tables are joined. |
| 3.training | transfer_from_mimic_plus_model_fusion_tuned.ipynb | In this notebook GCP TPU(s) are loaded; Madrid’s previously cleaned training and test CXR jpg(s) and EHR data are loaded; then we put images, EHR data and the labels in the same tf records; EHR data only model is defined; CXR only model is defined; pretrained MIMIC CXR model to identify CheXpert 14 labels is loaded; intermediated fusion model is defined; models are tuned; experiments are set up in order to find the best parameters; CXR model is retrained on full dataset using best parameters; finally results are visualized. |
|  | madrid_ehr_and_fusion_4fold.ipynb | In this notebook the code is used to train the EHR-based model, CXR-based model fusion model inMadrid data. Hyper-parameters were tuned using a 4-fold cross validation approach. The results of 4-fold internal validation were also computed. |
| 4.testing | hoboken_test_3models.ipynb | In this notebook the code is used to validate the pretrained EHR-based model, CXR-based model fusion model in the Hoboken dataset. |
|  | seoul_test_3models.ipynb | In this notebook the code is used to validate the pretrained EHR-based model, CXR-based model fusion model in the Seoul dataset. |

# **References**

[1] J. Mongan, L. Moy, and C. E. Kahn, “Checklist for Artificial Intelligence in Medical Imaging (CLAIM): A Guide for Authors and Reviewers,” *Radiol. Artif. Intell.*, vol. 2, no. 2, p. e200029, Mar. 2020, doi: 10.1148/ryai.2020200029.

[2] P.-C. Kuo *et al.*, “Recalibration of deep learning models for abnormality detection in smartphone-captured chest radiograph,” *NPJ Digit. Med.*, vol. 4, no. 1, p. 25, Feb. 2021, doi: 10.1038/s41746-021-00393-9.
